# Cost-effectiveness of inclisiran in patients with atherosclerotic cardiovascular disease from Chinese healthcare perspective

**DOI:** 10.1371/journal.pone.0350294

**Published:** 2026-05-28

**Authors:** Mochou Liu, Xinyue Zhang, Mengwen Feng, Qingfeng He, Zhen Feng

**Affiliations:** 1 Department of Pharmacy, the Affiliated Hospital of Xuzhou Medical University, Xuzhou, Jiangsu, China; 2 School of Public Health, Fudan University, Shanghai, China; 3 Department of Cardiology, the Affiliated Hospital of Xuzhou Medical University, Xuzhou, Jiangsu, China; 4 Department of Clinical Pharmacy and Pharmacy Administration, School of Pharmacy, Fudan University, Shanghai, China; Integral University, INDIA

## Abstract

**Background:**

Atherosclerotic cardiovascular disease (ASCVD) imposes a substantial clinical and economic burden in China. Despite maximally tolerated statin therapy, many patients fail to achieve recommended low-density lipoprotein cholesterol (LDL-C) targets. Inclisiran, a novel small interfering ribonucleic acid (siRNA) therapy, provides sustained LDL-C reduction, but its economic value under the Chinese healthcare system remains uncertain.

**Objective:**

To evaluate the cost-effectiveness of inclisiran when added to standard lipid-lowering therapy for patients with ASCVD in China.

**Methods:**

A Markov cohort multistate-transition model was developed from the perspective of the Chinese healthcare system, using a one-year cycle length and a lifetime horizon. Clinical inputs, costs, and utilities were derived from published literature and clinical trials. Both costs and outcomes were discounted at 4.5% annually. The primary result of the economic evaluation was the incremental cost-effectiveness ratio (ICER), with inclisiran considered cost-effective if the ICER was below the willingness-to-pay (WTP) threshold of CNY 191,498 per quality-adjusted life year (QALY), equivalent to two times China’s per capita gross domestic product in 2024. One-way and probabilistic sensitivity analyses were conducted to assess model uncertainty.

**Results:**

Compared with statin therapy alone, inclisiran added to statin therapy yielded an additional 0.41 QALYs and incurred incremental costs of CNY 58,321.35, resulting in an ICER of CNY 143,044.57/QALY, which is well below the WTP threshold. One-way sensitivity analyses showed that the mortality reduction effect, discount rate, and inclisiran price were the main drivers of model outcomes; however, inclisiran remained cost-effective across all tested scenarios. Probabilistic sensitivity analysis showed a 95.1% probability that inclisiran is cost-effective at the current price.

**Conclusions:**

At its current negotiated price of CNY 2,790 per dose, inclisiran added to standard therapy is cost-effective for patients with ASCVD in China.

## 1 Introduction

Atherosclerotic cardiovascular disease (ASCVD) is characterized by the buildup of lipid-rich plaques in the arterial wall, which may rupture and lead to acute coronary syndrome, angina, stroke, transient ischemic attack (TIA), and peripheral arterial disease [1]. In China, cardiovascular disease (CVD) remains the leading cause of death, accounting for 48.98% of deaths in rural areas and 47.35% in urban areas in 2021 [2]. The high incidence, recurrence, and mortality of ASCVD impose significant clinical and economic burdens on patients and society [3–5].

Elevated low-density lipoprotein cholesterol (LDL-C) is the most well-established modifiable risk factor for ASCVD, and lipid-lowering therapy significantly reduces cardiovascular (CV) events [6,7]. A meta-analysis conducted by the Cholesterol Treatment Trialists’ Collaboration (CTTC) demonstrated a 22% reduction in major vascular events per 1 mmol/L reduction in LDL-C [7]. International and Chinese guidelines emphasize stringent LDL-C targets for ASCVD patients [8–10]. The China Cholesterol Education Program (CCEP) recommends an LDL-C target of <1.8 mmol/L or a ≥ 50% reduction from baseline for high-risk individuals [10].

Despite the central role of statins in lipid-lowering therapy, many Chinese patients fail to reach LDL-C targets, even with high-intensity regimens. This is partly due to lower statin tolerability and a higher incidence of adverse effects among Chinese patients [11,12]. Among individuals at high ASCVD risk, 74.5% had uncontrolled LDL-C levels, yet only 5.5% received treatment [13]. This gap highlights the urgent need for novel lipid-lowering therapies.

Proprotein convertase subtilisin/kexin type 9 (PCSK9) regulates LDL-C metabolism by promoting degradation of LDL receptors, thereby raising circulating LDL-C levels [14]. PCSK9 inhibitors, including monoclonal antibodies, significantly reduce LDL-C and major adverse cardiovascular events (MACE) [8,9]. More recently, RNA interference (RNAi)-based therapies have emerged as a novel approach. Inclisiran, a small interfering RNA (siRNA) that inhibits hepatic PCSK9 synthesis, offers a convenient twice-yearly dosing regimen [15,16]. Clinical trials have demonstrated that inclisiran reduces LDL-C levels by approximately 50%, with sustained efficacy and good tolerability [17]. A pooled analysis of Phase III trials suggested a reduction in composite MACE (odds ratio [OR] 0.74), although effects on individual endpoints such as myocardial infarction and stroke were not statistically significant [18,19].

As healthcare costs rise, evaluating the cost-effectiveness of new therapies is critical for guiding policy and clinical decisions. International evidence shows that the economic value of inclisiran is highly price-sensitive, with studies from Australia, Switzerland, and the United States (US) finding it cost-effective only at substantially reduced prices [20–22].

In China, inclisiran was approved in 2023 as the first siRNA-based therapy for cardiovascular disease. However, its previously high cost and limited real-world evidence in Chinese populations created uncertainty about its economic value. Recently, national price negotiations reduced the price of inclisiran by more than 70%, and it has been included in the 2026 National Reimbursement Drug List (NRDL). To date, no study has evaluated its cost-effectiveness at this newly negotiated price.

This study aimed to evaluate the cost-effectiveness of inclisiran combined with statins versus statin alone in ASCVD patients in China, providing updated evidence to inform clinical decision-making and health policy.

## 2 Methods

### 2.1 Model structure

We developed a Markov cohort multistate-transition model from the perspective of the Chinese healthcare system with a lifetime horizon to simulate the disease progression and treatment outcomes for ASCVD patients. The model structure was adapted from previous cost-effectiveness evaluations of inclisiran [20–22]. The model includes six mutually exclusive health states: baseline ASCVD, non-fatal myocardial infarction (MI), non-fatal ischemic stroke (IS), post-MI, post-IS, and death (CV or non-CV). Patients enter the model in the baseline ASCVD state. Each annual cycle allows patients to remain in their current state or transition to another based on predefined probabilities. After a non-fatal MI or IS, patients transition to the corresponding post-event state in the following cycle. Only one CV event is allowed per cycle, though multiple events can occur over a patient’s lifetime.

**Fig 1:**
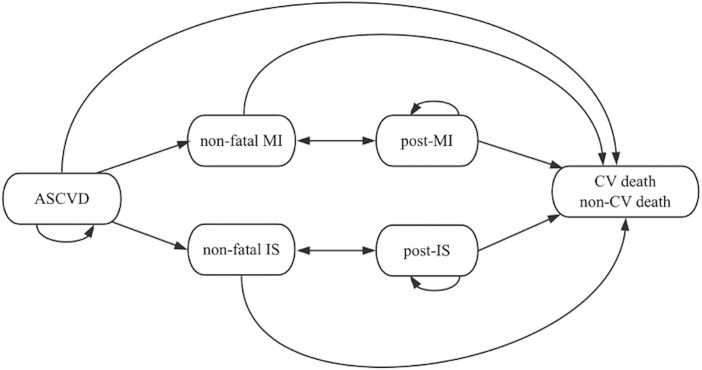
Markov model structure. Abbreviations: ASCVD atherosclerotic cardiovascular disease; CV cardiovascular; IS ischemic stroke; MI myocardial infarction.

The model assumes a memoryless process, where transition probabilities depend only on the current state, not prior events. The cycle length is one year. A simplified schematic of the model structure is shown in Fig 1.

The model was developed using TreeAge Pro Healthcare Version 2022 (TreeAge Software, LLC., Williamstown, MA, USA). Reporting followed the Consolidated Health Economic Evaluation Reporting Standards 2022 (CHEERS 2022) checklist (S1 Table) [23].

### 2.2 Target population and treatments

Baseline characteristics were derived from the ORION-18 trial, a phase 3, randomized, double-blind, placebo-controlled study conducted in China, South Korea, Singapore, and Taiwan [24]. The trial enrolled adults with ASCVD and elevated LDL-C (≥1.8 mmol/L) or at high risk of ASCVD. The mean age was 59.5 years, and 74.7% were male, and 74.7% were Chinese, making the population representative of clinical practice in China.

Two treatment strategies were compared in the model. The intervention strategy was inclisiran added to standard lipid-lowering therapy, while the comparator strategy was standard lipid-lowering therapy alone. In the ORION-18 trial, patients in the intervention group received 300 mg of inclisiran administered subcutaneously on days 1, 90, and 270 in addition to background lipid-lowering therapy, with 3.2% receiving low-intensity statins, 27.6% moderate-intensity, and 69.2% high-intensity. Patients in the comparator group received statin therapy alone. Baseline LDL-C levels were 108.8 mg/dL in the inclisiran group and 109.1 mg/dL in the statin alone group [24]. These comparators were selected to reflect the current standard of care for secondary prevention in ASCVD patients in China and to evaluate the incremental clinical and economic value of adding inclisiran to existing lipid-lowering therapy.

### 2.3 Baseline cardiovascular event rates

Baseline CV event rates (a composite of MI, IS, and CV death) were sourced from the US Truven MarketScan database, which reported 6.40 events per 100 patient-years in an ASCVD cohort with a mean LDL-C level of 2.96 mmol/L and a mean age of 57 years [25]. To account for the higher CV risk observed in Asian populations, we first adjusted this rate using a hazard ratio (HR) of 1.04 [26–28], resulting in an initial Asian-adjusted rate of 6.66 events per 100 patient-years.

Further adjustments were then applied to account for differences in age and LDL-C levels between the US cohort and Chinese population in the ORION-18 study. Adjustments were performed using Equation 1 [28]:


$$\begin{array}{c} r_{a}=r_{\text{0}}\times \overset{\Delta age}{\underset{age}{HR}}\times {RR}^{\Delta LDLc} \end{array}$$ (1)

where $r_{a}$ is the adjusted CV event rate for the Chinese population; $r_{\text{0}}$ is the baseline event rate; ${HR}_{age}$ is the hazard ratio per year of age from the re-estimated REACH model [29]; Δage is the age difference between the Chinese and and the US cohorts; RR is the rate ratio per 1 mmol/L reduction in LDL-C (0.78; 95% credible interval [CI]: 0.76–0.80) based on the CTTC meta-analysis; ΔLDLc is the difference in LDL-C (mmol/L) between the two cohorts.

Annual event-specific rates for MI, IS, and CV death were further disaggregated according to the distribution of outcomes reported in a pooled meta-analysis of the ORION-9/10/11 [30]. To account for increased risk following a previous CV event, we applied risk multipliers from the re-estimated REACH models [29] (Table 1). Finally, all event rates were converted into transition probabilities using the standard exponential formula (Equation 2) [31]:

**Table 1:** Key inputs in the model.

Parameter	Value	Range	Distribution	Source
Patient characteristics			Fixed	[24]
Mean age (years)	59.5	NA		
Male (%)	74.7	NA		
Mean LDL-C level (mg/dl)	109.0	NA		
Statin usage (%)	97.7	NA		
Baseline CV event rates (per 100 patient-years)			Fixed	Calculated
Composite CV event	6.66	(5.33, 7.99)		
MI	3.53	(2.82, 4.24)		
IS	1.72	(1.38, 2.06)		
CV death	0.70	(0.56, 0.84)		
HRs for increased CV risk due to prior events			Lognormal	[29]
Recurrent MI	1.13	(1.04, 1.22)		
Recurrent IS	1.13	(0.99, 1.30)		
RR per 1 mmol/L reduction in LDL-C			Lognormal	[7]
MACE	0.78	(0.76, 0.80)		
MI	0.73	(0.70, 0.76)		
IS	0.79	(0.74, 0.85)		
CV death	0.84	(0.80, 0.88)		
All cause death	0.90	(0.87, 0.93)		
Effect of intervention			Normal	[24]
LDL-C reduction (%)	57.2	(51.3, 63.1)		
Absolute LDL-C reduction (mmol/L)	1.56	(1.50, 1.63)		
Annual costs (CNY)			Gamma	[32,33]
^a^ Inclisiran (year 1)	8,385.0	(6,708, 8,385.0)		
Inclisiran (subsequent years)	5,590.0	(4,472.0, 5,590.0)		
Statin therapy	3,645.0	(2,916.0, 3,645.0)		
MI (year 1)	23,567.4	(18,853.9, 28,280.9)		
MI (subsequent years)	3,885.2	(3,108.2, 4,662.2)		
IS (year 1)	10,005.7	(8,004.6, 12,006.8)		
IS (subsequent years)	4,398.0	(3,518.4, 5,277.6)		
CV death	15,212.0	(12,169.6, 18,254.4)		
Non-CV death	6,044.1	(4,835.3, 7,252.9)		
Health utilities			Beta	[34,35]
Baseline ASCVD	0.824	(0.800, 0.848)		
MI (year 1)	0.672	(0.625, 0.719)		
MI (beyond 1 year)	0.824	(0.800, 0.848)		
IS (year 1)	0.327	(0.264, 0.390)		
IS (beyond 1 year)	0.524	(0.472, 0.576)		

^a^Include an injection fee of CNY 5 per administration. Abbreviations: ASCVD atherosclerotic cardiovascular disease; CNY Chinese yuan; CV cardiovascular; HR hazard ratio; IS ischemic stroke; LDL-C low-density lipoprotein cholesterol; MACE major cardiovascular event; MI myocardial infarction; NA not applicable; RR rate ratio.


$$\begin{array}{c} P=\text{1}-exp(-rt) \end{array}$$ (2)

where P is the annual transition probability; r is the event rate; t is the cycle length (1 year).

### 2.4 Treatment effects

In the ORION-18, inclisiran reduced LDL-C by 57.2% relative to placebo (absolute change: –60.5 mg/dL, *p* < 0.001) [24]. Since ORION-18 did not report CV outcomes, we estimated treatment effects on CV event rates using the CTTC meta-analysis, which established a well-validated relationship between LDL-C reduction and reductions in CV events. This method is widely used in cost-effectiveness analyses of lipid-lowering therapies, including PCSK9 inhibitors.

According to the CTTC meta-analysis, the rate ratio (RR) for major vascular events was 0.78 (95% CI: 0.76–0.80) per 1 mmol/L reduction in LDL-C [7]. Event-specific RRs were 0.73 (95% CI: 0.70–0.76) for MI, 0.79 (95% CI: 0.74–0.85) for IS, and 0.84 (95% CI: 0.80–0.88) for CV death [7] (Table 1).

Annual post-treatment event rates for the inclisiran group were calculated using Equation 3 [28,36]:


$$\begin{array}{c} r_{tx}=r_{\text{0}}\times {RR}^{\Delta LDLc} \end{array}$$ (3)

where $r_{tx}$ is the event rate after treatment; $r_{\text{0}}$ is the baseline event rate; RR is the rate ratio per 1 mmol/l LDL-C reduction; ΔLDLc is the absolute LDL-C reduction (mmol/L). The resulting event-specific annual rates were then converted to transition probabilities to reflect the risk of CV events in patients receiving inclisiran plus background statin therapy.

### 2.5 Mortality

All-cause mortality was derived from age-specific mortality rates in the general Chinese population using data from the 2023 China Health Statistics Yearbook (S2 Table) [32], and used as the baseline mortality in the model. CV mortality rates were extracted from the same source, and non-CV mortality was calculated by subtracting CV mortality from the total all-cause mortality.

To reflect the increased risk of death following a cardiovascular event, patients who experienced an MI or IS in the preceding year were assigned a higher risk of CV death, based on a hazard ratio of 1.31 (95% CI: 1.15–1.49) [37]. This adjustment ensures that mortality estimates capture both background population risk and the additional event-specific mortality risk.

### 2.6 Costs and utilities

From the perspective of the Chinese healthcare system, only direct medical costs, including medications and costs associated with modeled CV events, were considered, while indirect costs were excluded due to data limitations and uncertainty. Total costs were calculated by multiplying the costs of each health state by the probability of occupying that state over the model’s time horizon.

The updated list price of inclisiran is CNY 2,790 per dose, as reported by the Chinese drug procurement platform. An injection fee of CNY 5 per dose was added, based on local tertiary hospital charges. Inclisiran was administered as a 300 mg subcutaneous injection at days 1 and 90, followed by once every six months. Accordingly, the annual treatment cost was CNY 8,370 in the first year and CNY 5,580 in subsequent years. Statins prices were derived from the maximum prices under Volume-Based Procurement (VBP) policy in China [38]. Daily treatment costs were estimated according to the labeled therapeutic doses for low-, moderate-, and high-intensity statin regimens, as defined in clinical guidelines (S3 Table). To reflect real-world prescribing patterns, intensity-specific utilization proportions from the ORION-18 study [24] were applied to derive a weighted average daily cost of CNY 9.99, corresponding to an annual cost per patient of CNY 3,645 (S3 Table).

Annual treatment and hospitalization costs for each health state were derived from the 2023 China Health Statistics Yearbook and relevant domestic literature [32]. All costs were adjusted to 2024 levels using the Consumer Price Index (CPI) published by the National Bureau of Statistics [39] and are expressed in Chinese Yuan (Table 1).

Health state utility values were sourced from published time trade-off studies [34,35] (Table 1). Comparable utility estimates have been widely used in previous Chinese pharmacoeconomic evaluations of cardiovascular therapies [27,28,36], supporting the applicability of these values to the Chinese healthcare setting. Total quality-adjusted life years (QALYs) were calculated by accumulating health state utilities over each model cycle, weighted by the time spent in each health state and the corresponding state occupancy probabilities. A half-cycle correction was applied to both costs and QALYs to account for transitions occurring, on average, halfway through each annual cycle.

Both costs and utilities were discounted at an annual rate of 4.5%, based on recent empirical evidence indicating that a moderately lower discount rate more appropriately reflects current economic conditions in China [40].

### 2.7 Economic analyses

A lifetime horizon was adopted to fully capture the long-term effects of lipid-lowering therapy in chronic disease. Health benefits were expressed in QALYs gained, and the incremental cost-effectiveness ratio (ICER) was calculated as the ratio of incremental costs to incremental QALYs. An intervention was considered cost-effective if the ICER fell below the willingness-to-pay (WTP) threshold of CNY 191,498 per QALY, equivalent to two times China’s per capita gross domestic product (GDP) in 2024 [41], in accordance with recommendations from local pharmacoeconomic guidelines and the World Health Organization (WHO) [42].

### 2.8 Sensitivity analyses

Sensitivity analyses were conducted to assess the impact of parameter uncertainty and to evaluate the robustness of the model. In the one-way sensitivity analysis, individual parameters were varied within their lower and upper bounds, typically using 95% CIs or ±20% ranges when specific intervals were unavailable. The discount rate was varied from 0% to 5%, and drug costs were reduced by up to 20% to reflect potential future price decreases under China’s drug pricing policy. Key variables included treatment costs, utility values, inclisiran efficacy, and RRs for major CV events (Table 1). Results were presented using Tornado diagrams to identify influential variables.

Probabilistic sensitivity analysis (PSA) was performed using 1,000 Monte Carlo simulations. Input distributions followed standard statistical distributions: gamma for costs, lognormal for RRs, normal for mean LDL-C reduction, and beta for transition probabilities and utilities. Results were visualized using scatter plots and cost-effectiveness acceptability curves (CEACs).

Scenario analyses were conducted to assess the impact of treatment duration by applying alternative time horizons ranging from 5 to 30 years.

### 2.9 Model validation

The model was adapted from previously published and validated economic evaluation frameworks for PCSK9 inhibitors and underwent face and internal validation, though no independent external validation was performed. Face validity was confirmed through expert review by clinical pharmacists and health economists, who assessed the model structure, assumptions, inputs, and outputs for clinical relevance and logical consistency. Internal validity was assessed through systematic checks of data sources, formulas, and computations, with independent cross-verification of the model code and results confirming correct implementation.

## 3 Results

### 3.1 Base case analyses

In the base case analysis, adding inclisiran to statin therapy resulted in a gain of 0.41 QALYs and an incremental cost of CNY 58,321.35 compared with statin alone, yielding an ICER of CNY 143,044.57 per QALY gained (Table 2). This value is well below the WTP threshold of CNY 191,498 per QALY, equivalent to two times China’s per capita GDP in 2024, indicating that inclisiran is cost-effective at its current price.

**Table 2:** Base case cost-effectiveness results of inclisiran vs. statin alone in Chinese ASCVD patients.

Treatment strategy	Total cost（CNY）	Health outcomes（QALYs）	Incremental cost（CNY）	Incremental QALYs	ICER（CNY/QALY）
Statin	83,140.31	11.18	NA	NA	NA
Inclisiran	141,461.65	11.59	58,321.35	0.41	143,044.57

Abbreviations: CNY Chinese yuan; ICER incremental cost-effectiveness ratio; NA Not applicable; QALY quality-adjusted life year.

### 3.2 Sensitivity analyses

**Fig 2:**
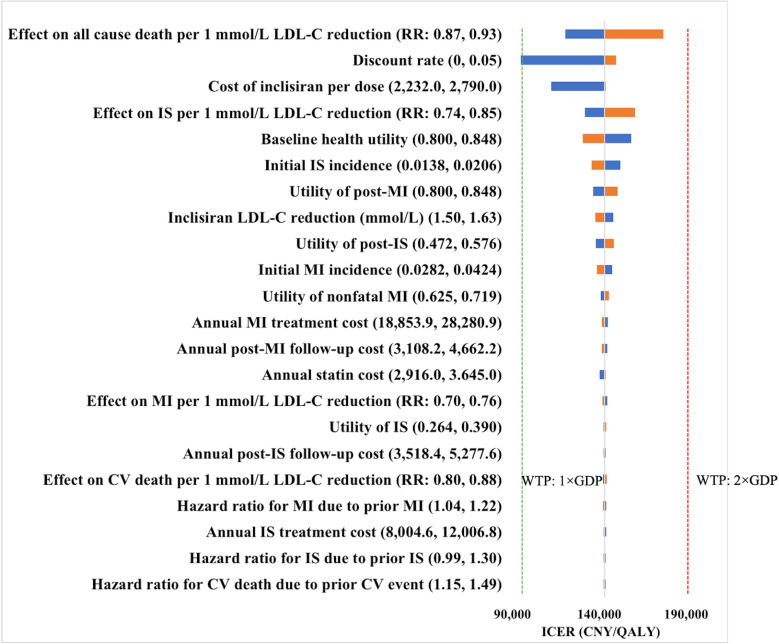
Tornado diagram comparing inclisiran added to statin vs. statin alone. Blue bars indicate ICERs at lower parameter values, and yellow bars indicate ICERs at upper parameter values. The green dotted line indicates the WTP threshold of one times GDP per capita (CNY 95,749/QALY), and the red dotted line indicates the WTP threshold of two times GDP per capita (CNY 191,498/QALY). Abbreviations: CV cardiovascular; IS ischemic stroke; LDL-C low-density lipoprotein cholesterol; MI myocardial infarction.

In the one-way sensitivity analysis, the ICER was most sensitive to the effect on all-cause mortality per l mmol/L reduction in LDL-C (ICER range: CNY 120,282.64–176,656.87/QALY), the discount rate (CNY 94,569.79–149,456.49/QALY), and the per-dose cost of inclisiran (CNY 112,138.81–143,044.57/QALY). Across all scenarios, the ICER remained below the WTP threshold, confirming the cost-effectiveness of inclisiran under current assumptions (Fig 2).

**Fig 3:**
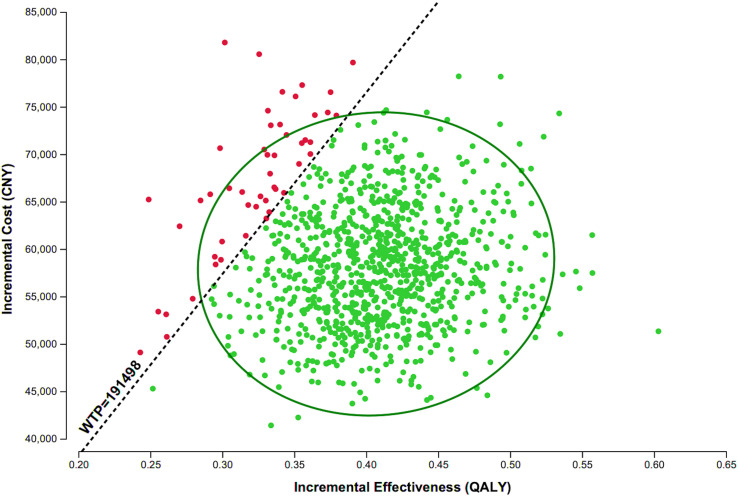
Incremental cost-effectiveness scatter plot. Each dot represents a simulated ICER. The dashed line indicates the WTP threshold of CNY 191,498/QALY. Abbreviations: QALY, quality-adjusted life years; WTP, willingness-to-pay.

**Fig 4:**
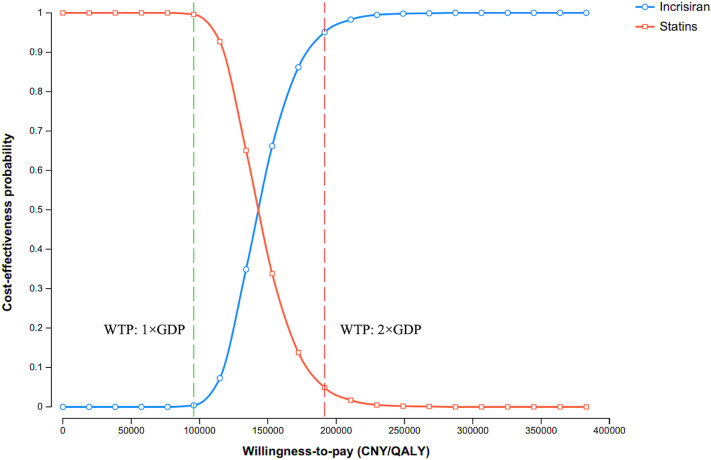
Cost-effectiveness acceptability curve. The green dotted line denotes the WTP threshold of one times GDP per capita (CNY 95,749/QALY), and the red dotted line denotes the WTP threshold of two times GDP per capita (CNY 191,498/QALY). Abbreviations: GDP, gross domestic product.

In the PSA, 95.1% of simulated ICERs fell below the WTP threshold of CNY 191,498/QALY, indicating a high probability that inclisiran is cost-effective at its current price (Fig 3). The CEAC shows that the probability of cost-effectiveness was 0.4% at a threshold of one times GDP per capita (CNY 95,749/QALY) and increased to 95.1% at two times GDP per capita (CNY 191,498/QALY) (Fig 4).

### 3.3 Scenario analysis

As the time horizon increased from 5 to 30 years, both incremental costs and QALYs gradually increased. Correspondingly, the ICER decreased substantially over time, from CNY 865,929.95/QALY at 5 years to CNY 143,044.57/QALY at 30 years (Table 3). These results indicate that the cost-effectiveness of inclisiran improves over longer time horizons, with the ICER falling well below the WTP threshold of CNY 191,498/QALY under ≥25 years horizon.

**Table 3:** Cost-effectiveness results of inclisiran vs. statin alone across different time horizons.

Time horizon (years)	Incremental cost（CNY）	Incremental QALYs	ICER（CNY/QALY）
5	25,554.76	0.03	865,929.95
10	40,002.68	0.09	445,140.76
15	48,992.70	0.16	298,624.64
20	54,292.15	0.25	221,224.03
25	57,123.62	0.33	173,539.06
30	58,321.35	0.41	143,044.57

Abbreviations: CNY Chinese yuan; ICER incremental cost-effectiveness ratio; QALY quality-adjusted life year.

## 4 Discussion

This study developed a lifetime Markov model from the perspective of China’s healthcare system to evaluate the cost-effectiveness of inclisiran in patients with ASCVD under the current nationally negotiated price. At a per-dose price of CNY 2,790, inclisiran added to statin therapy yielded an ICER of CNY 143,044.57 per QALY, which is well below the commonly accepted WTP threshold in China. These findings indicate that, following substantial price reductions, inclisiran is cost-effective in the Chinese healthcare setting.

Our findings differ from earlier Chinese economic evaluations, such as the study by Zhou et al. [43], which concluded that inclisiran was not cost-effective in China. Their analysis was conducted under a substantially higher assumed per-dose price (CNY 20,000), resulting in an ICER of CNY 2,127,756 per QALY, and they estimated that a price reduction to approximately CNY 2,500 per dose would be required to achieve cost-effectiveness. Importantly, the current nationally negotiated price of inclisiran (CNY 2,790 per dose) closely aligns with this threshold, explaining the change in cost-effectiveness conclusions. Differences in ICER estimates across studies can also be attributed to variations in model structure, input parameters, and assumptions regarding long-term outcomes.

International evidence consistently highlights the strong price sensitivity of inclisiran’s economic value. Studies from Australia, Switzerland, and the United States have shown that inclisiran was not cost-effective at initial launch prices but became economically favorable only after substantial price reductions [20–22]. The present analysis demonstrates that China’s national price negotiation mechanism has similarly reshaped the value proposition of inclisiran, bringing its ICER into an acceptable range under local WTP thresholds.

A comparable pattern has been observed for other PCSK9 inhibitors in China. Liang et al. found that evolocumab was not cost-effective at its original price (CNY 1,298 per dose) for Chinese patients with myocardial infarction [44]. After national price negotiations reduced the price to CNY 283.8 per dose and included it in the NRDL, the ICER fell into an acceptable range [28]. These experiences suggest that centralized price negotiations and reimbursement inclusion are effective tools for improving patient access to high-value cardiovascular therapies while maintaining budgetary sustainability.

Nevertheless, sustained reliance on large price cuts may hinder pharmaceutical innovation [45]. Value-based pricing (VBP) offers a more balanced approach by aligning drug prices with clinical benefits while incorporating innovation, safety, affordability, and societal expectations [46,47]. With China’s dynamic NRDL adjustment process, integrating VBP into reimbursement decision-making could support evidence-based, sustainable access to innovative therapies [48]. Future pricing of inclisiran should consider production cost, clinical and economic value, system affordability, and expert appraisal.

Sensitivity analyses identified the effect on all-cause mortality per 1 mmol/L LDL-C reduction as the most influential driver of ICER estimates. Mendelian randomization studies show lifelong LDL-C reductions are associated with proportional decreases in coronary mortality for lipid-lowering therapies [49]. Consistent with prior evolocumab cost-effectiveness studies [28,35], our model linked LDL-C reductions observed in ORION-18, a representative Chinese population, to cardiovascular risk reductions from the CTTC meta-analysis. While ORION-18 did not report MACE outcomes, pooled analyses from ORION-9, −10, and −11 trials showed a relative risk of 0.76 for MACE, though all-cause mortality reductions were not statistically significant [18], supporting the plausibility of our assumptions while highlighting some uncertainty in long-term mortality effects. Sensitivity analyses also identified the discount rate as a key driver of ICERs. This finding is consistent with pharmacoeconomic theory and prior studies emphasizing the importance of discounting in long-term evaluations of chronic disease [50–52]. Because the cardiovascular benefits of LDL-C lowering accrue gradually over time, higher discount rates disproportionately reduce the present value of health gains. International guidelines recommend discount rates ranging from 1.5% to 5%, and some suggest lower rates for health outcomes to avoid undervaluing long-term benefits [53].

Patient risk stratification is another important factor influencing cost-effectiveness. Evidence suggests that the absolute benefit of LDL-C reductions increases with baseline LDL-C levels and ASCVD risk [54]. In high-risk US patients, Fonarow et al. [55] showed that evolocumab achieved ICERs below the local WTP threshold. Targeting high-risk subgroups could enhance the value of inclisiran and avoid unnecessary expenditure [56]. However, differences in drug prices and reimbursement systems limit the transferability of these findings to China. Our model did not incorporate risk stratification, which may underestimate cost-effectiveness among very high-risk patients. Future studies should evaluate risk-based strategies tailored to Chinese populations.

This study has several limitations. First, the ORION trials reported outcomes over a median follow-up of 18 months, whereas our model extrapolated benefits over a lifetime, potentially overestimating long-term effectiveness. Second, although the baseline population characteristics were primarily derived from the ORION-18 trial, in which 74.7% of participants were Chinese and therefore broadly representative of Chinese clinical practice, several model inputs, including transition probabilities and utility values, were sourced from international studies due to limited availability of China-specific data. In particular, cardiovascular event reductions associated with inclisiran were inferred using CTTC meta-analysis estimates based on LDL-C lowering effects rather than direct long-term cardiovascular outcome data in Chinese patients. Additionally, some utility parameters were derived from international studies; however, these estimates were based on validated preference-based methods and have been widely adopted in previous Chinese pharmacoeconomic evaluations of cardiovascular therapies. Extensive sensitivity analyses further demonstrated the robustness of the model results. Third, the model assumed perfect adherence, without accounting for real-world discontinuation, likely overstating QALY gains. Fourth, only direct medical costs were included; indirect costs and societal benefits, such as productivity gains, were excluded. Injection-site reaction costs were not modeled, though such events are typically mild and self-limiting. Future research should incorporate Chinese real-world data, evaluate alternative reimbursement strategies, and explore cost-effectiveness in high-risk ASCVD subgroups.

## 5 Conclusions

At the current nationally negotiated price of CNY 2,790 per dose, inclisiran added to standard therapy for ASCVD is cost-effective in China under commonly accepted willingness-to-pay thresholds. This represents a substantial shift from earlier evaluations conducted at higher prices and highlights the critical role of national price negotiations in improving the economic value and accessibility of innovative cardiovascular therapies. These findings provide timely, model-based evidence to support clinical decision-making and reimbursement policy for inclisiran in the Chinese healthcare system.

## Supporting information

S1 Table Consolidated health economic evaluation reporting standards 2022 (CHEERS 2022) checklist. (DOCX)

S2 Table Age-specific annual probabilities of cardiovascular and non-cardiovascular death in the general Chinese population, based on mortality rates by age and cause of death from the 2023 China Health Statistics Yearbook. (DOCX)

S3 Table Cost of statin therapy. (DOCX)
