# Supplementary material for: Cost-effectiveness of inclisiran in patients with atherosclerotic cardiovascular disease from Chinese healthcare perspective
Source: PLoS One. 2026 May 28;21(5):e0350294. doi: 10.1371/journal.pone.0350294 (PMC13218505; doi:10.1371/journal.pone.0350294)
Supplement: S2 Table — (DOCX) [file pone.0350294.s002.docx]

**S2Table. Age-specific annual probabilities of cardiovascular and non-cardiovascular death in the general Chinese population, based on mortality rates by age and cause of death from the 2023 China Health Statistics Yearbook.**

| Age | Death probability (%) | | |
| --- | --- | --- | --- |
|  | All-cause death | CV death | Non-CV death |
| 55-59 | 0.0044 | 0.0007 | 0.0038 |
| 60-64 | 0.0072 | 0.0011 | 0.0061 |
| 65-69 | 0.0121 | 0.0019 | 0.0102 |
| 70-74 | 0.0199 | 0.0035 | 0.0164 |
| 75-79 | 0.0336 | 0.0067 | 0.0271 |
| 80-84 | 0.0581 | 0.0138 | 0.0449 |
| ≥85 | 0.1391 | 0.0428 | 0.1007 |
