# Supplementary material for: Cost-effectiveness of inclisiran in patients with atherosclerotic cardiovascular disease from Chinese healthcare perspective
Source: PLoS One. 2026 May 28;21(5):e0350294. doi: 10.1371/journal.pone.0350294 (PMC13218505; doi:10.1371/journal.pone.0350294)
Supplement: S3 Table — (DOCX) [file pone.0350294.s003.docx]

**S3 Table.** **Cost of statin therapy**

| Generic name | ^a^ Low-intensity (CNY/day) | ^b^ Moderate-intensity (CNY/day) | ^c^ High-intensity (CNY/day) |
| --- | --- | --- | --- |
| Atorvastatin | - | 6.1 | 12.2 |
| Rosuvastatin | - | 5.5 | 11.0 |
| Pitavastatin | 2.6 | 10.2 | - |
| Simvastatin | 1.1 | 4.5 | - |
| Pravastatin | 2.1 | 8.2 | - |
| Fluvastatin | 1.7 | 6.8 | - |
| Average price (CNY/day) | 1.88 | 6.88 | 11.6 |
| Weighted average cost (CNY/day) | 9.99 | | |
| Annual cost per patient (CNY) | 3,645.0 | | |

Notes: ^a^ Low-intensity: pitavastatin 1 mg, fluvastatin 20 mg, simvastatin 10 mg, pravastatin 10 mg.

^b^ Moderate-intensity: atorvastatin 20 mg, rosuvastatin 10 mg, pitavastatin 4 mg, simvastatin 40 mg, pravastatin 40 mg, fluvastatin 80 mg.

^c^ High-intensity: atorvastatin 40 mg, rosuvastatin 20 mg.

Drug costs were derived from average maximum prices in volume-based procurement (VBP) in China ^[1]^.

The weighted daily cost was calculated as the sum of (daily cost × proportion of patients on each statin intensity).

The distribution of statin intensity was obtained from the ORION-18 study: low = 3.2%, moderate = 27.6%, high = 69.2% ^[2]^.

Annual cost = weighted daily cost × 365 days.

References:

[1] Yaozhi database. https://www.yaozh.com/. Accessed 13 May, 2025.

[2] Huo Y, Lesogor A, Lee CW, et al. Efficacy and Safety of Inclisiran in Asian Patients: Results From ORION-18. JACC Asia. 2024;4(2):123-134.
